# Managing suicide risk in primary care

**DOI:** 10.1192/bjb.2021.95

**Published:** 2021-12

**Authors:** Ann Maria Albert, Hannah Gallen, Misha Gaur

**Affiliations:** GPST3, Oxford GPVTS, UK. Email: Annmaria.albert1@nhs.net; GPST1, Reading GPVTS, UK; GPST3, Reading GPVTS, UK

05 June 2021

We read with interest Professor Morgan's special article on predicting short-term suicide risk.^1^ We are grateful for the mention of the extensive body of evidence suggesting the futility of suicide risk assessments and alleged risk factors including suicidal thoughts and behaviours in predicting suicide risk. We appreciate the statements ‘To base assessment of ongoing risk on the individual's mental state during a single interview is clearly likely to be highly unreliable’ and ‘An important trigger for relapse is stress, particularly stress that has previously been present and has not been resolved’. It is important that the above facts are conveyed to the patient's general practitioner (GP) via the suggested correspondence. However, we wonder about the purpose of the proposed 123-word paragraph ending with the sentence ‘Overall, however, the predicted level of suicide risk must still be regarded as significant, requiring vigilance until I next see him/her’. What action is required of the GP when they receive similar letters about almost every patient seen by the mental health services? If the patient requires vigilance for their mental health, would this not best be provided by secondary care mental health services with their array of highly specialist teams and army of experts? What aspects of suicide prevention are the GPs better equipped for than the secondary care mental health services? It is important to acknowledge that it is not possible to reliably predict suicide risk from single consultations. However, it appears the suggested correspondence is unrealistically asking an already overstretched primary care service to pick up responsibility in a specialist area. Furthermore, we would be grateful for any guidance on how to better assess and manage suicide risk during a 10 min GP consultation than during the 30–60 min assessment by specialists.
